# Vitamin D Is Required for ILC3 Derived IL-22 and Protection From *Citrobacter rodentium* Infection

**DOI:** 10.3389/fimmu.2019.00001

**Published:** 2019-01-22

**Authors:** Yang-Ding Lin, Juhi Arora, Kevin Diehl, Stephanie A. Bora, Margherita T. Cantorna

**Affiliations:** ^1^Department of Veterinary and Biomedical Science, The Pennsylvania State University, Pennsylvania, PA, United States; ^2^Center for Molecular Immunology and Infectious Disease, The Pennsylvania State University, Pennsylvania, PA, United States; ^3^Cedars-Sinai Medical Center, Los Angeles, CA, United States

**Keywords:** vitamin D, ILC3, IL-22, gastrointestinal infection, Th17

## Abstract

*Citrobacter rodentium* is a gastrointestinal infection that requires early IL-22 from group 3 innate lymphoid cells (ILC3) for resistance. The role of vitamin D in the clearance of *C. rodentium* infection was tested in vitamin D sufficient (D+) and vitamin D deficient (D-) wildtype (WT) and Cyp27B1 (Cyp) KO mice (unable to produce the high affinity vitamin D ligand 1,25(OH)_2_D, 1,25D). Feeding Cyp KO mice D- diets reduced vitamin D levels and prevented synthesis of 1,25D. D- (WT and Cyp KO) mice had fewer ILC3 cells and less IL-22 than D+ mice. D- Cyp KO mice developed a severe infection that resulted in the lethality of the mice by d14 post-infection. T and B cell deficient D- Rag KO mice also developed a severe and lethal infection with *C. rodentium* compared to D+ Rag KO mice. D- WT mice survived the infection but took significantly longer to clear the *C. rodentium* infection than D+ WT or D+ Cyp KO mice. Treating infected D- Cyp KO mice with IL-22 protected the mice from lethality. Treating the D- WT mice with 1,25D reconstituted the ILC3 cells in the colon and protected the mice from *C. rodentium*. IL-22 treatment of D- WT mice eliminated the need for vitamin D to clear the *C. rodentium* infection. Vitamin D is required for early IL-22 production from ILC3 cells and protection from enteric infection with *C. rodentium*.

## Introduction

Vitamin D primarily functions as a regulator of calcium homeostasis and plays a critical role in bone formation and resorption. The discovery of the vitamin D receptor (VDR) in cells of the immune system and the presence of the vitamin D 1α-hydroxylase (Cyp27B1; Cyp) in dendritic cells and macrophage suggested that locally-produced 1,25(OH)_2_D (1,25D) has regulatory autocrine and paracrine properties in the immune system (1, 2). The vitamin D 1α-hydroxylase (Cyp27B1) converts the circulating form of vitamin D, 25(OH)D, to the high affinity VDR binding form, 1,25D [Supplementary Figure 1, 3]. 25(OH)D is a low affinity ligand of the VDR, that in the absence of 1,25D will bind and regulate vitamin D target genes [Supplementary Figure 1, 4]. The VDR bound to 1,25D acts as a transcription factor to regulate the expression of genes with vitamin D response elements in their promoters (5). Vitamin D metabolism is tightly regulated by a feed-back loop that includes 1,25D/VDR as an inducer of the vitamin D 24-hydroxylase (Cyp24A1) that eliminates the VDR binding activity of both 25(OH)D and 1,25D (6). Cyp27B1 KO mice accumulate 25(OH)D when they are fed vitamin D containing diets, because of a failure to induce Cyp24A1 [Supplementary Figure 1, 4]. The VDR is constitutively expressed in most immune cells (7, 8). Resting T cells and B cells express low levels of the VDR, which are upregulated following activation (8, 9). Taken together, it is clear that the immune system is a vitamin D target.

1,25D has been shown to suppress Th1/Th17 responses and diseases including experimental inflammatory bowel disease (IBD) 10. Vitamin D deficiency (D-) and VDR deficiency (knockout, KO) exacerbated experimental IBD in multiple models of colitis (11–13). The decrease in IL-17 and IFN-γ production and the increase in T reg cells caused by 1,25D both *in vitro* and *in vivo* have been suggested as mechanisms underlying the ability of 1,25D to suppress IBD (14–16). Because of the inhibitory effects of 1,25D on Th1 and Th17 cells, infections that require Th1/Th17 cell responses for resistance might be more severe in 1,25D treated mice. However, 1,25D had no effect on the ability of mice to fight either a *Candida albicans* or a *Herpes simplex* virus infection (17). 1,25D injections inhibited Th17 responses and increased *Citrobacter rodentium* bacterial shedding at d10 post-infection (18). Conversely, VDR KO mice were more susceptible to infections with *Listeria monocytogenes, Salmonella* and *C. rodentium* than wildtype (WT) mice (19, 20). 1,25D treatment either had no effect or increased susceptibility to infection, while VDR deficiency increased susceptibility to several different pathogens including *Salmonella* and *C. rodentium*. The contradictory effects of 1,25D vs. VDR deficiency on infection may be a result of differential effects of vitamin D on inflammation vs. clearance.

*C. rodentium* is a gram-negative murine bacterial pathogen, and a model of human infection with enterohemorrhagic and enteropathogenic *Escherichia coli* infections. WT mice clear *C. rodentium* infection within 4–5 weeks of infection (21). CD4+ T cells, B cells, as well as T-dependent IgG responses are required for clearance of *C. rodentium* (21). IL-22 KO mice develop a lethal infection with *C. rodentium* and IL-22 from ILC3 cells is required for early protection from *C. rodentium* (22–24). Th17 cells and production of IL-17 and IL-22 are needed for the host to clear *C. rodentium* infection (25). In sum, Th17 and ILC3 cells are critical for host resistance to *C. rodentium*.

Here the effect of vitamin D status on mucosal immunity to *C. rodentium* was determined. Mice that could not produce 1,25D (Cyp27B1 knockout, Cyp KO) were used to model severe vitamin D deficiency (Supplementary Figure 1, D- Cyp KO). In the D- Cyp KO mice, the residual 25(OH)D is not present at high enough concentrations to bind the VDR (Supplementary Figure 1). D- Cyp KO mice and D- Rag KO mice developed a severe *C. rodentium* infection that resulted in early lethality of the mice. D- WT mice produced some of the high affinity 1,25D ligand from the residual 25(OH)D (Supplementary Figure 1). D- WT mice took longer to clear a *C. rodentium* infection than vitamin D sufficient (D+) WT and D+ Cyp KO mice. There was no difference in the susceptibility of D+ WT and D+ Cyp KO mice to *C. rodentium*. D- mice had reduced numbers of ILC3 cells that produced IL-22 in the colon compared to D+ mice. In addition, the expansion of colonic Th17 cells that make IL-17 and IL-22 were lower following *C. rodentium* infection in D- mice. 1,25D treatment of D- mice recovered ILC3 cells and increased the rate of *C. rodentium* clearance compared to D- mice. Treatment of D- Cyp KO mice with IL-22 prevented the lethality and treatment of D- WT mice with IL-22 eliminated the need for vitamin D in the clearance of *C. rodentium*. Vitamin D is required for ILC3 derived IL-22 in the colon and the induction of protective Th17 cell expansion following infection. In the absence of vitamin D, mice develop a more severe enteric infection that takes longer to resolve.

## Methods

### Mice

C57BL/6 CD19Cre, LckCre, Rag1 KO, and B6.129P2(Cg)- Rorc^tm2Litt/J^ (Rorc^gfp^) mice were originally from the Jackson Laboratories (Bar Harbor, ME) and bred for experiments at the Pennsylvania State University (University Park, PA). C57BL/6 Cyp27B1 (henceforth described as Cyp KO) KO breeders were a gift from Dr. Hector DeLuca (University of Wisconsin, Madison, WI). Rorc^gfp^ mice were crossed with Rag1 KO mice to generate Rorc^gfp^ Rag1 KO mice for cell sorting experiments of intestinal ILCs. VDR^fl/fl^ mice were a gift from Dr. S. Kato (University of Tokyo, Tokyo, Japan) and were crossed with CD19Cre (B-VDR KO) or LckCre (T-VDR KO) mice. VDR^fl/fl^ Cre+ (B-VDR KO or T-VDR KO) and their corresponding VDR^fl/fl^ Cre- littermates (designated as WT) were used for experiments. Mice were fed purified diets made in the lab as described previously that differed only in vitamin D content (26). To generate D- and D+ mice, breeders were fed diets with or without vitamin D throughout breeding and continued on the same diets throughout the experiments. Experimental mice were Cyp KO and WT littermates from the same D+ or D- breeders (Supplementary Figure 1, 26. The vitamin D status (25(OH)D levels) of the offspring were measured prior to doing experiments and serum 25(OH)D levels of 10–20ng/mL were used as the cut-off for vitamin D deficiency (26). For some experiments, 1,25D was added to the D- diets as described (26). The 1,25D dose was 25 ng/d for mice that weighed 10–18g and 50 ng/d when the mice weighed >18g as described previously (26). Some mice received IL-22Fc injections intraperitoneally (100 μg of IL-22Fc (Genentech, South San Francisco, CA) or 100 μg of IgG2a isotype control (LTF-2, BioXCell, West Lebanon, NH) twice a week beginning at d3 post-infection for 2 weeks and a total of 4 doses per mouse as described previously (27). All of the experimental procedures were approved by the Institutional Animal Care and Use Committee at the Pennsylvania State University (University Park, PA).

### Serum 25(OH)D Measurements

Serum 25(OH)D levels were measured by colorimetric assay using a 25(OH)D ELISA Kit (Eagle Biosciences, Nashua, NH), according to manufacturer's instructions. The limit of detection was 1.6 ng/mL 25(OH)D.

### *C. rodentium* Infection

The *C. rodentium* strain ICC169 (nalidixic acid resistant) was a gift of Dr. Gad Frankel (London School of Medicine and Dentistry, London UK). *C. rodentium* was cultured overnight in Luria-Bertani broth with 50 μg/ml nalidixic acid (EMD chemicals, Gibstown, NJ). Mice were orally gavaged with 5 × 10^9^ colony forming units (CFU) of *C. rodentium* in PBS. In some experiments, one dose of vancomycin (20 mg/mL, MP Biomedicals, Solon, OH) was given by oral gavage the day before *C. rodentium* infection. The vancomycin pretreatment increased infectivity without affecting the shedding kinetics of *C. rodentium* (20). Feces and tissue samples were collected, weighed, homogenized, and serially diluted and plated to determine the CFU (28). Histopathology of the distal colon was scored blinded on a scale from 0 to 4 (0 = none; 1 = minimal; 2 = mild; 3 = moderate; 4 = extensive) for inflammation and epithelial hyperplasia as previously described (29, 30). Total histology scores were the sum of the inflammation and epithelial hyperplasia scores for a total score from 0 to 8.

### *C. rodentium*-Specific Antibody Responses

Detection of *C. rodentium*-specific IgA, IgG1, and IgG2c was done by coating plates with 30 μg/ml sonicated *C. rodentium* overnight. Ig were detected using goat anti-mouse detection antibodies and reported as relative units *C. rodentium* specific IgA (Bethly Laboratories), IgG1and IgG2c (BD Biosciences).

### Cell Isolation and Flow Cytometry

Colon LP cells were isolated as described previously and stained for flow cytometry (20). Briefly, the whole colon was washed, cut into 1-1.5 cm sections, incubated twice in Hanks' Balanced Salt Solution (HBSS, Sigma-Aldrich, St. Louis, MO) with 5mM EDTA at 37°C and then digested in RPMI-1640 containing 1 mg/ml collagenase type 1 (Worthington, Lakewood, NJ) and 10% FBS at 37°C for 1h in a shaking incubator. The cells were collected from the interface of 40/80% Percoll gradients (Sigma-Aldrich). The fluorescent dye conjugated-antibodies listed below were used for flow cytometry: Anti-CD3 (145-2C11), CD4 (GK1.5) (Biolegend, San Diego, CA), Thy1.2 (30-H12), CD45.2 (104) (BD Biosciences, San Jose, CA), FoxP3 (FJK-16s), RORγt (B2D), IL-22 (IL22JOP), IL-17A (TC11-18H10) (eBioscience, San Diego, CA). For intracellular cytokine staining, cells were stimulated with PMA (0.1 μg/ml, Sigma-Aldrich), ionomycin (0.5 μg/ml, Sigma-Aldrich) in the presence of Brefeldin A (10 μg/ml, Sigma-Aldrich) for 4 h. For IL-22 intracellular staining, mouse recombinant IL-23 (40 ng/ml, R&D systems, Minneapolis, MN) was added to the PMA, ionomycin and Brefeldin A cultures for 4 h. The cells were fixed and permeabilized using kits for intracellular staining (eBioscience) according to the manufacturer's instructions. All data were collected on a BD Fortessa LSRII (BD Biosciences) and analyzed with FlowJo software (TreeStar, Ashland, OR).

### Real-Time PCR

A 1 cm sample of the distal colon was used to measure *hprt*, β*-actin, ifn-*γ*, il-17a, il-22, vdr*, and *rorc* by real-time PCR. Total RNA was isolated from 1 cm distal colons of mice using RNeasy Mini kit (Qiagen, Valencia, CA). cDNA was synthesized by using AMV reverse transcriptase (Promega, Madison, WI) and analyzed using the StepOnePlus real-time PCR system (Thermo Fisher Scientific, Waltham, MA). Gene expression was normalized to *Hprt* or β*-actin* amplified and calculated with the ΔΔCt method. The primer sequences are listed in Supplementary Table 1.

### Statistics

Statistical analyses were performed with PRISM software by (GraphPad, La Jolla, CA). Two-tailed Student's *t* test or two-way ANOVA with Bonferroni *post-hoc* tests were used for data analyses as indicated. The survival rates were compared by log-rank tests. For all the analyses, *P* values (^*^ < 0.05 ^**^ < 0.01, ^***^ < 0.001, ^****^ < 0.0001) were used to indicate significance. Values are the means ± SEM.

## Results

### Vitamin D Deficient Mice Have Increased Susceptibility to *C. rodentium* Infection

**Figure 1:**
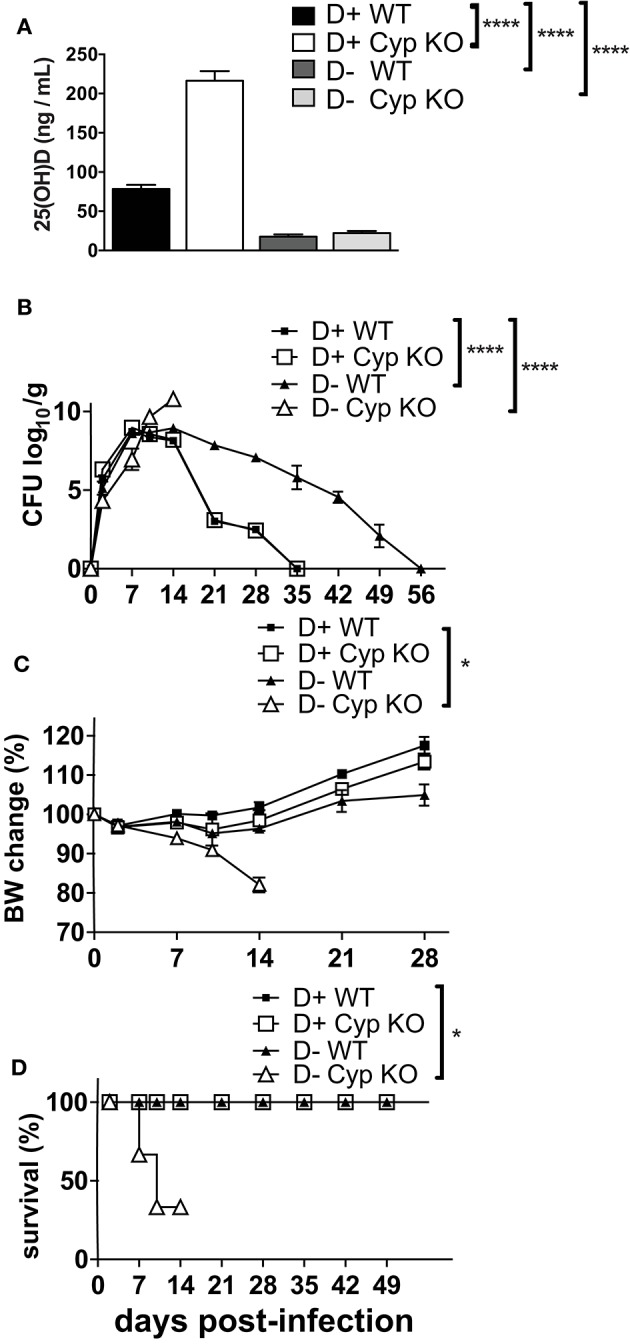
Increased susceptibility of D- mice to *C. rodentium* infection. **(A)** Serum 25(OH) D levels in 8 week-old D**+** WT, D+ Cyp KO, D- WT, and D- Cyp KO mice. Values are the mean ± SEM of a total *n* = 6–13 mice/group. Mice were infected with *C. rodentium* and **(B)** fecal shedding of the pathogen was monitored, **(C)** the percent original body weight (BW), and **(D)** the survival rate were plotted vs. days post-infection. The values **(B–D)** represent the mean ± SEM of *n* = 3–5 mice/group and from one representative of two independent experiments. One-way ANOVA with Bonferroni *post-hoc* tests **(A)**, Two-way ANOVA with Bonferroni *post-hoc* tests **(B, C)** and log-rank test **(D)** were used to determine significance. ^*^P < 0.05, ^****^P < 0.0001.

There are contradictory effects of 1,25D and VDR deficiency on host resistance to *C. rodentium* (18, 20). Here we determined the effects of dietary vitamin D deficiency on *C. rodentium* susceptibility. Cyp27B1 is the enzyme that produces high affinity 1,25D from the precursor 25(OH)D (Supplementary Figure 1). Feeding Cyp KO mice D+ diets results in the accumulation of 25(OH)D in the serum, because of a failure to induce Cyp24A1 (Supplementary Figures 1, 4). As expected, the 25(OH)D concentration in the D+ Cyp KO mice was significantly higher than in the D+ WT mice fed the same D+ diets (Figure 1A). The kinetics of *C. rodentium* infection in D+ Cyp KO and D+ WT mice were indistinguishable with a peak of infection at d7-14 and then clearance by d35 (Figure 1B). D- mice had serum 25(OH)D values < 20 ng/mL that confirmed their D- status (Figure 1A). The D- WT mice had low 25(OH)D levels (Supplementary Figure 1, Figure 1A) in the serum that could still be made into some of the high affinity 1,25D. The D- Cyp KO mice had low 25(OH)D (Supplementary Figure 1, Figure 1A) and were unable to produce any 1,25D that resulted in the complete absence of VDR binding ligands and severe vitamin D deficiency. D- Cyp KO mice developed an acute *C. rodentium* infection that resulted in a significant loss of body weight and premature death of the D- Cyp KO mice (Figures 1B–D). The surviving D- Cyp KO mice were sacrificed (33%) at d14, because of excessive body weight loss (Figure 1D). D- WT mice shed significantly more *C. rodentium* at d21 post-infection than D+ mice (Figure 1B). The D- WT mice took significantly longer to clear the infection than the D+ mice (Figure 1B). Vitamin D deficiency increased the susceptibility of mice to *C. rodentium* infection. At d14 post-infection D+ (Supplementary Figure 2A) mice had no *C. rodentium* in the spleen or liver, while the amount of *C. rodentium* was significantly higher in both D- WT and D- Cyp KO mice (Supplementary Figure 2A). Because there were no differences in the D+ Cyp KO and D+ WT kinetics of *C. rodentium* clearance, future experiments combined the groups into one D+ group. For D- mice there were no differences in the histopathology scores between WT and Cyp KO mice at d14 post-infection (Cyp KO sections shown in Supplementary Figure 2B). Histopathology of the colons from D- mice showed severe inflammation, epithelial hyperplasia and higher histopathology scores than the D+ mice at d14 post-infection (Supplementary Figure 2C). Severely vitamin D deficient D- Cyp KO developed a fulminating and lethal disease following *C. rodentium* infection.

### Impaired *C. rodentium*-Specific Antibody Production in D- Mice

**Figure 2:**
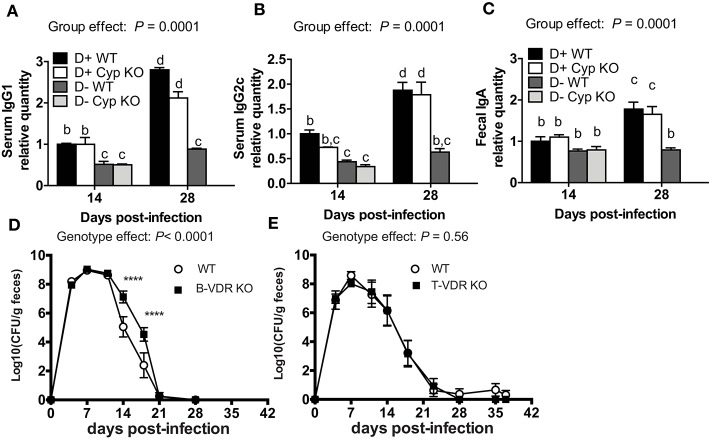
Indirect roles of vitamin D in B cells and T cells for clearance of *C. rodentium*. The amount of *C. rodentium*-specific **(A)** IgG1 and **(B)** IgG2c in the serum. Fecal **(C)** *C. rodentium* specific IgA. D+ WT values at d14 were set at 1 and other values are relative to the D + WT value. Values are from two independent experiments and the mean ± SEM of *n* = 6–8 mice per group. Two-way ANOVA with Bonferroni *post-hoc* tests **(A–C)**. Groups without a common letter differ at the indicated time point, *P* < 0.05. Fecal shedding of *C. rodentium* in **(D)** WT and B-VDR KO or **(E)** WT and T-VDR KO littermates following *C. rodentium* infection. Values are the mean ± SEM of two combined experiments and *n* = 9–13 mice per group. Significance was determined using two-way ANOVA with Bonferroni *post-hoc* tests. ^****^P < 0.0001.

The antigen specific antibody response was measured to determine whether antibodies could explain the increased susceptibility of D- mice to *C. rodentium* infection. D+ WT and D+ Cyp KO mice mounted a *Citrobacter*-specific IgG1 and IgG2c response that was detectable at d14 and increased by d28 post-infection (Figures 2A,B). D- WT and D- Cyp KO mice had very low *Citrobacter*-specific IgG1 and IgG2c responses at both d14 and d28 post-infection (Figures 2A,B) The IgG1 or IgG2c *C. rodentium*-specific response was similar in D+ WT and D+ Cyp KO mice at either d14 or d28 post-infection (Figures 2A,B). D- (WT and Cyp KO) mice had significantly lower *Citrobacter*-specific IgG1 and IgG2c titers than the D+ mice at d14 and d28 post-infection (Figures 2A,B). There was no increase in the antibody titers from d14 to d28 in D- mice (Figures 2A,B). *C. rodentium*-specific fecal IgA levels were not different at d14 in the D+ and D- mice (Figure 2C). The fecal IgA levels went up at d28 post-infection in D+ mice but not in D- mice (Figure 2C). D- mice have reduced *Citrobacter*-specific antibody responses compared to D+ mice.

In order to determine whether T cells or B cells were the direct targets of vitamin D, mice with the VDR deleted in B cells (B-VDR KO) or T cells (T-VDR KO) were infected with *C. rodentium*. The shedding curves of B-VDR KO mice and their WT littermates were similar until d14-d18 when the B-VDR KO mice had more *C. rodentium* shed in the feces than WT (Figure 2D). There was a significant effect of genotype on the shedding kinetics in WT and B-VDR KO mice (Figure 2D). However, WT and B-VDR KO mice both cleared *C. rodentium* by d21 post-infection (Figure 2D). The *C. rodentium*-specific IgG1 and IgG2c responses at d14 and d28 post-infection were not different in the WT and B-VDR KO littermates (data not shown). In the WT and T-VDR KO littermates there was no effect of genotype on the shedding of *C. rodentium* (Figure 2E). There was no effect of deleting the VDR in T cells and a small effect (higher bacterial loads) of deleting the VDR in B cells on the ability of mice to clear *C. rodentium* infection. The data suggest that vitamin D is not directly regulating T or B cell mediated immune responses to *C. rodentium*.

### Higher IL-17 mRNA in the Colons From D- Mice at d14 Post-infection

**Figure 3:**
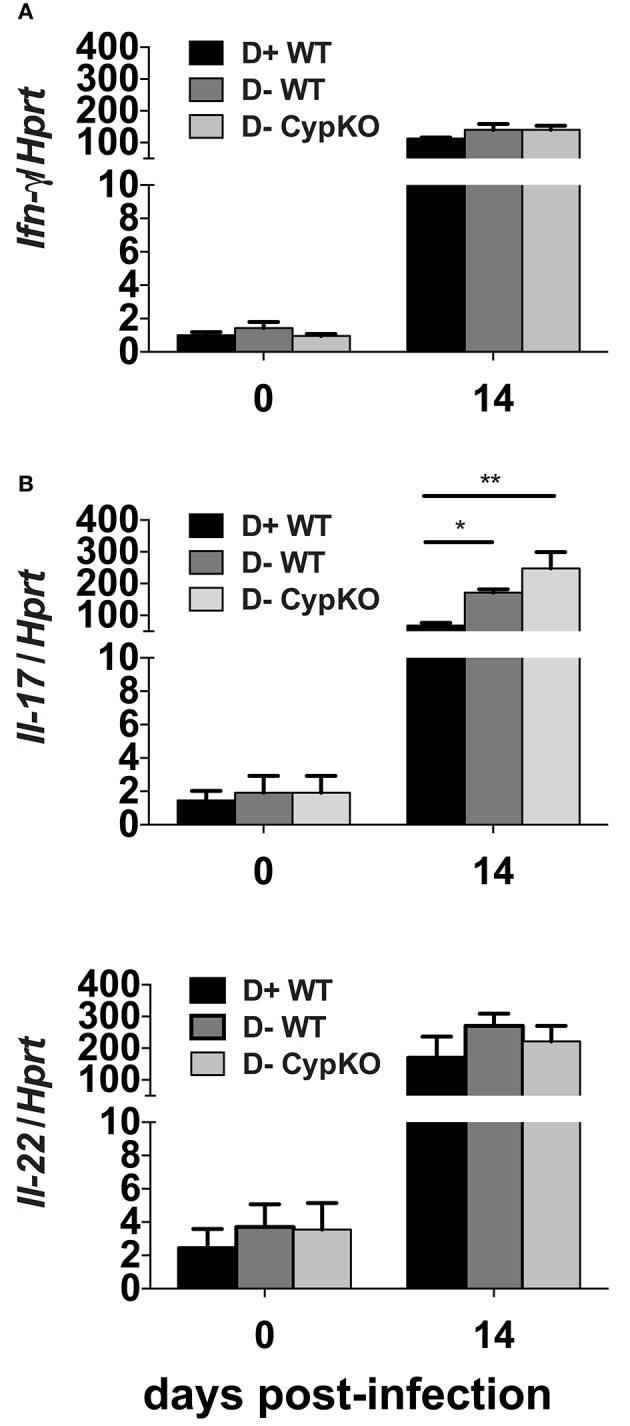
Increased expression of IL-17 mRNA in D- colons at d14 post-infection. The expression of mRNA for **(A)** *il-22*, **(B)** *il-17*, and **(C)** *ifn-*γ relative to *hprt* in the distal colon of mice. Values are the mean ± SEM of *n* = 3–5 colons/group and one representative of two independent experiments. Statistical analysis was performed by two-way ANOVA with Bonferroni *post-hoc* tests. ^*^P < 0.05, ^**^P < 0.01.

The distal colon was used as a source of mRNA to determine the effect of vitamin D on several cytokines important for host resistance to *C. rodentium*. Because there were no differences in *C. rodentium* susceptibility between D+ WT and D+ Cyp KO mice, the D+ Cyp KO group was eliminated. Before infection, the mRNA levels for *ifn-*γ, *il-17*, and *il-22* were low and not different in D+ WT, D- WT, and D- Cyp KO mice (Figure 3). Infection increased expression of the mRNA for *ifn-*γ, *il-17*, and *il-22* at d14 post-infection in all mice (significant time effect Figures 3A–C). *Ifn-*γ and *il-22* were not different between D+ WT, D- WT and D-Cyp KO mice at d14 post-infection (Figures 3A,C). At d14 post-infection, *il-17* was significantly lower in D+ WT mice than in D- WT mice (*P* = 0.008) and D- Cyp KO (*P* < 0.0001) mice (Figure 3B). D- WT *il-17* mRNA expression was lower than D- Cyp KO *il-17* mRNA in the d14 infected colon (*P* = 0.08, Figure 3B). D- mice had higher amounts of *il-17* mRNA in the distal colon compared to D+ mice.

### Fewer ILC3 and Th17 Cells and Less IL-22 and IL-17 in the Colon of D- Mice

**Figure 4:**
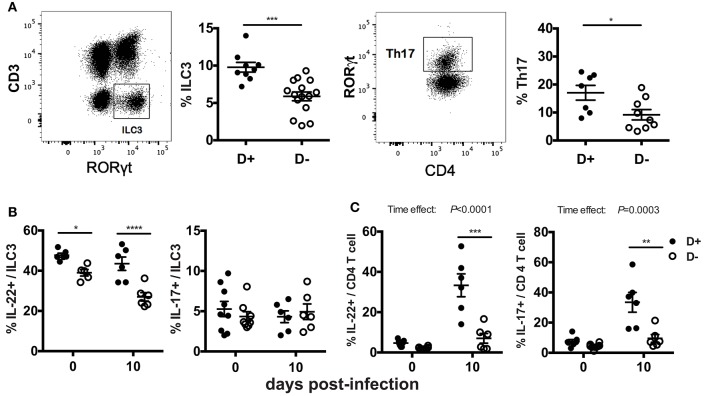
Fewer ILC3 and Th17 cells in the colon of D- mice. Gating examples and frequencies of **(A)** ILC3 (Thy1+RORγt+/CD3-) and Th17 (CD4+RORγt+/CD3+) in the colon of D+ and D- mice. The frequencies of **(B)** ILC3 and **(C)** Th17 cells that secrete IL22 (left) or IL-17 (right). Values are mean ± SEM of two combined experiments and **(A)** ILC3, *n* = 9–15; Th17, *n* = 7–11, and **(B)** *n* = 6–9 mice per group. Significance was determined using two-tailed Student's *t* tests **(A)**, two-way ANOVA with Bonferroni *post-hoc* tests **(B,C)**. ^*^P < 0.05, ^**^P < 0.01, ^***^P < 0.001, ^****^P < 0.0001.

The mRNA expression for *il-17* and *il-22* in the distal colon may not represent protein expression throughout the colon. In addition, the cellular source of the IL-17 and IL-22 is unclear in the mRNA analyses in Figure 3. ILC3 cells and Th17 cells that produce IL-17 and IL-22 express RORγt and are critical for clearance of *C. rodentium* (22–25). ILC3 and Th17 cells were measured in D+ and D- colon LP. The total numbers of cells, the frequency of T cells, TCRβ+, TCRβ+/CD4+, TCRβ+/CD8+, and TCRβ+/CD8αα were not different in the colon as a result of vitamin D status or genotype (data not shown). ILC3 frequencies (Thy-1+/RORγt+/CD3-) and Th17 frequencies (CD4+/RORγt+/CD3+/FoxP3-) were significantly higher in D+ as compared to D- colon (Figure 4A). D- mice had fewer ILC3 in both the SI and colon (Figure 4A, Supplementary Figure 3). The frequencies of Th17 cells were also higher in D+ than D- mice (Figure 4A). The number of IL-22 secreting ILC3 cells in the whole colon were higher in D+ than D- mice at d0 and d10 post-infection (Figure 4B). The frequencies of IL-17 secreting ILC3s, or Th17 were similar at d0 in D+ and D- colons (Figures 4B,C). Infection significantly increased the frequencies of T cells that made IL-22 and IL-17 (significant time effect, Figure 4C), but infection had no effect on the frequencies of ILC3 cells that made IL-22 and IL-17 (Figure 4B). IL-22 secreting ILC3s were higher in D+ than D- colon before and after infection (Figure 4B). T cells that made IL-17 and IL-22 were higher in the D+ vs. D- colon following infection (Figure 4C). The mean fluorescence intensity (MFI) of IL-22 was higher in D+ mice (compared to D- mice) at d10 post-infection in both the ILC3 and T cells (Supplementary Figures 4A,B). Conversely, the IL-17 MFI was significantly lower in the D+ ILC3 than D- ILC3 cells but not different in the D+ and D- T cells (Supplementary Figure 4A). D- mice had reduced numbers of IL-22 producing ILC3s and T cells in the whole colon than D+ mice. Conversely, in the distal colon there was no effect of vitamin D on mRNA for *il-22* (Figure 3C). The discrepancy in IL-22 results is likely due to sampling of only part of the colon in Figure 3 and the whole colon in Figure 4. In addition, the colon of D- mice had fewer ILC3 cells that made more IL-17 than the ILC3 cells in the D+ mice (Supplementary Figure 4A). Overall the data show that D- mice had less IL-22 from both ILC3 and T cells and less IL-17 from T cells than D+ mice.

### D- Rag KO Mice Are Highly Susceptible to *C. rodentium* Infection

**Figure 5:**
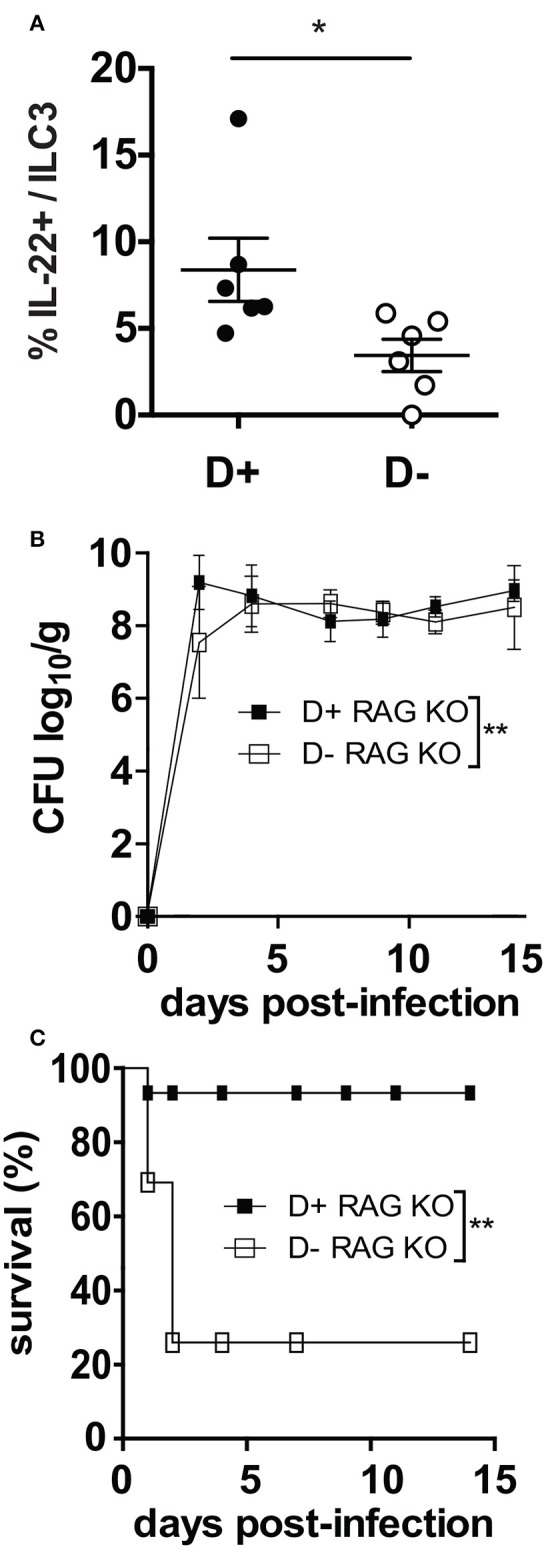
D- Rag KO mice develop a severe and lethal *C. rodentium* infection. **(A)** IL-22 secreting ILC3 frequencies in the colon of D- and D+ Rag KO mice. **(B)** *C. rodentium* fecal shedding in D+ and D- Rag KO mice. **(C)** Survival curves following *C. rodentium* infection of D+ and D- Rag KO mice. Values are from *n* = 6–7 D+ Rag KO and *n* = 6 D- Rag KO mice of two combined experiments. Significance was determined by two-tailed Student's *t* tests **(A)**, two-way ANOVA with Bonferroni *post-hoc* tests **(B)** or log rank tests for **(C)**, ^*^P < 0.05, ^**^P < 0.01.

ILC3 cells that produce IL-22 are critical in the survival and protection of Rag KO mice from *C. rodentium* (23). D- Rag KO mice had fewer IL-22 secreting ILC3 cells in the colon than D+ Rag KO (Figure 5A). By d2 post-infection both the D+ and D- Rag KO mice were shedding extremely high numbers of *C. rodentium* in the feces (Figure 5B). Only 1 out of 7 infected D+ Rag KO mice died from the infection within the first few days of infection (Figure 5C). Conversely, 4 of 6 infected D- Rag KO mice died from the infection within the first several days (Figure 5C). The mice that died had high numbers of *C. rodentium* in the liver and spleen, confirming that the death was due to the infection. Lethality was significantly higher in D- than D+ Rag KO mice (Figure 5C, *P* < 0.03).

### 1,25D or IL-22 Treatments Protect D- Mice From *C. rodentium* Infection

**Figure 6:**
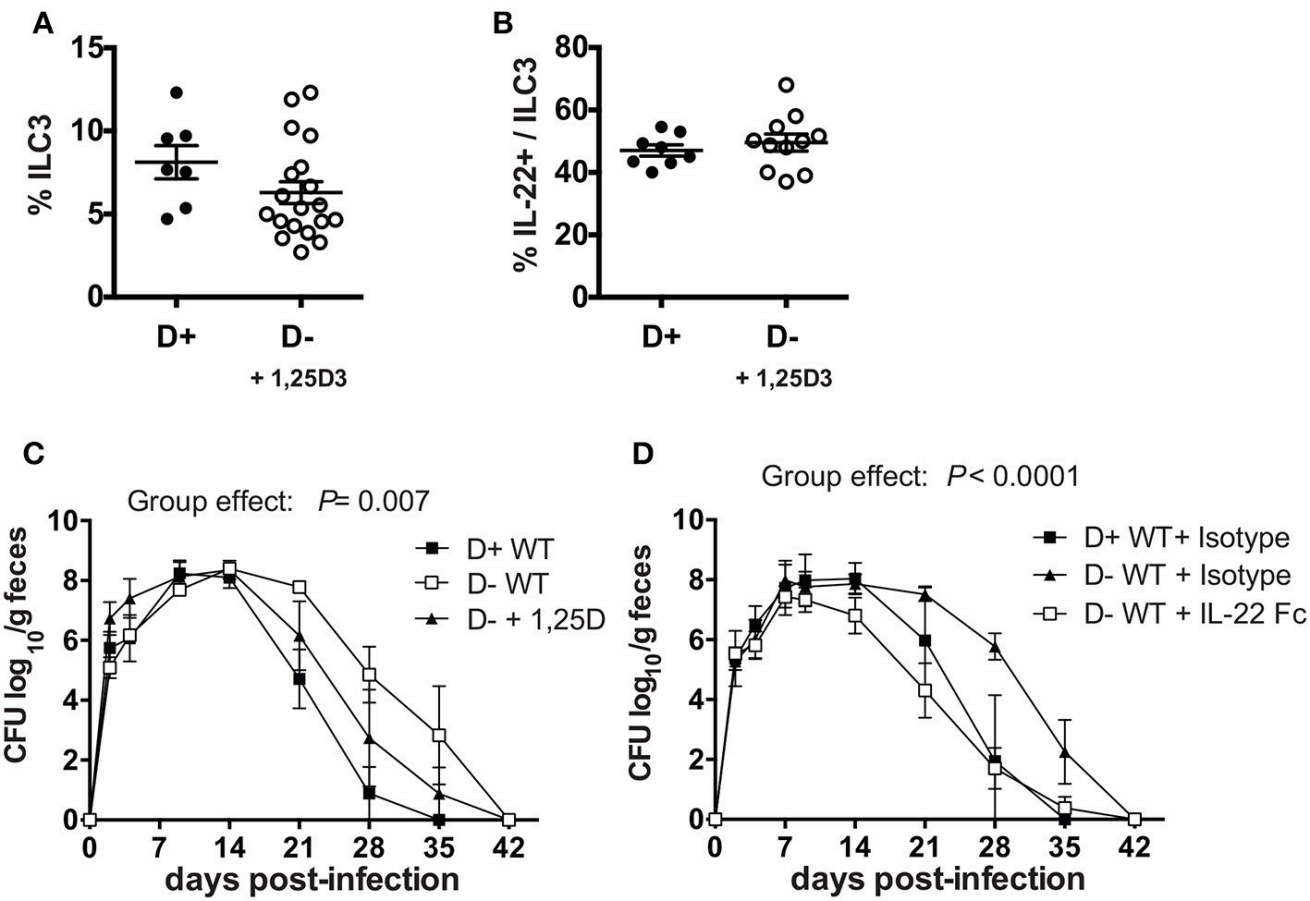
1, 25D treatment and IL-22 treatment of D- mice resulted in more rapid clearance of *C. rodentium* infection. **(A)** Frequencies of ILC3 cells in the colon of D- mice treated with 1, 25D. **(B)** Frequency of IL-22 secreting ILC3 cells following 1, 25D treatment. *C. rodentium* fecal shedding in D- WT mice treated with **(C)** 1,25D (D- + 1,25D) or **(D)** IL-22 treated (D- + IL-22Fc). Values are mean ± SEM of two combined experiments and **(A)** *n* = 7–19 **(B)** *n* = 8–11 **(C)** *n* = 4 **(D)** *n* = 6–9 mice per group. Significance was determined using two-tailed Student's *t* tests **(A,B)**, two-way ANOVA with Tukey *post-hoc* tests **(C,D)**.

Experiments were done to determine whether ILC3, and/or IL-22 were important for the protective effects of vitamin D in host resistance to *C. rodentium* infection. ILC3 cells express higher levels of mRNA for the VDR than other intestinal ILCs (Supplementary Figure 4C). To determine whether 1,25D treatments *in vivo* could recover the ILC3 cell numbers, 1,25D treatments were started in D- mice at 6 weeks of age (Supplementary Figure 4D) or at 3 weeks of age (Figure 6A). D- ILC3 frequencies were significantly lower than D+ values when the 1,25D treatment was started at 6 weeks of age (Supplementary Figure 4D), but reached D+ ILC3 values when the 1,25D treatment was started at 3 weeks of age (Figure 6A). The 1,25D treatment increased frequencies of ILC3 that made IL-22 in D- mice (Figure 6B). Infection of the 1,25D treated D- (D- + 1,25D) mice resulted in *C. rodentium* shedding kinetics that were significantly different than both D+ WT and D-WT mice (significant group effect, Figure 6C). Treatment of D- mice with an IL-22Fc fusion protein resulted in *C. rodentium* shedding kinetics that were more similar to D+ isotype treated than D- isotype treated mice (Figure 6D). Furthermore, the D+ WT and D-WT mice that received IL-22 Fc cleared the infection by d35 when the D- WT isotype treated mice still had 2 logs of *C. rodentium* in the feces (Figure 6D). Treating D- Cyp KO mice with IL-22 Fc protected the mice from early lethality (100% survival to d14 post infection) as compared to D- Cyp KO isotype treated mice (data not shown). 1,25D treatment increased IL-22 secreting ILC3 cells in D- mice to D+ levels and improved clearance of *C. rodentium* (Figure 6). IL-22 treatment eliminated the effect of vitamin D deficiency on *C. rodentium* clearance (Figure 6).

## Discussion

The increased susceptibility of D- mice to *C. rodentium* infection was due to reduced frequencies of colonic ILC3 and ILC3-derived IL-22. IL-22 treatment of D- WT mice eliminated the need for vitamin D for the clearance of *C. rodentium*. In addition, treating D- Cyp KO mice with IL-22 prevented the lethality of the mice from *C. rodentium*. IL-22 secreting ILC3 are required in Rag KO mice for early survival from *C. rodentium* (23). The majority of D- Rag KO mice (67%) died within two days of *C. rodentium* infection as compared to only 14% of D+ Rag KO mice. IL-22 is a protective cytokine that regulates epithelial tight junction proteins to enhance gut epithelial integrity and induces antimicrobial factors in the small intestine to control the microbiota (31–33). D+ and D- mice have distinct microbial communities, which could be due to reduced IL-22 in D- mice (34). Vitamin D regulates IL-22 producing ILC3 cells in the intestine.

ILC3 cells are vitamin D targets in the mouse colon. VDR mRNA levels were higher in mouse RORγt+ ILC3 cells from the colon than in other ILC cell populations (Supplementary Figure 4C). 1,25D treatment of D- mice beginning at 3wks of age was effective for increasing the frequency of ILC3 cells, but starting at 6 weeks of age was not. The timing of the 1,25D mediated effect either suggests that ILC3 cells in the colon are indirect vitamin D targets or that there may be a developmental requirement for vitamin D in ILC3 cells. ILC3 cells in the colon peak by 4wks of age to the levels found in 8 week old mice (35). ILC3 cells require several transcription factors including T-bet, Notch, Ahr, and RORγt for development (36). Of the transcription factors important for ILC3 development, there is some evidence that Notch may be a vitamin D target. Vitamin D deficient rats had reduced Notch signaling that resulted in muscle atrophy and decreased myocyte regeneration (37). The mechanisms by which 1,25D regulates ILC3 and IL-22 could involve induction of Notch, direct regulation of IL-22, regulation of ILC3 development and expansion of ILC3 subsets. These signals by which 1,25D regulates ILC3 cell numbers and the production of IL-22 in the colon should be explored further.

Without antibiotic pretreatment, VDR KO mice were resistant to colonization with *C. rodentium* (20). The colonization resistance of VDR KO mice was shown to correlate with the presence of more ILC3 cells in the SI [(20) and Supplementary Figure 3]. VDR KO and WT mice had similar frequencies of ILC3 cells in the colon [(20) and Supplementary Figure 3]. The data suggest that ILC3 cells in the colon vs. the SI might be differentially regulated by vitamin D and expression of the VDR. The VDR is a nuclear transcription factor that interacts with co-regulatory proteins in the nucleus. The VDR protein-protein interactions are not dependent on the presence of the vitamin D ligand. Others have shown differences between VDR KO and D- effects in other cell types (38). In the absence of its ligand, the VDR protein binds β-catenin and Lef1 proteins to form a complex that is involved in Wnt signaling (38). The vitamin D deficient cells would have the normal protein-VDR interactions, while the VDR KO cells would be missing the co-regulatory functions of the unliganded VDR. Nonetheless, the fact that both the VDR KO and D- ILC3 cell numbers change compared to D+ WT ILC3 suggests a critical role of the VDR and vitamin D in ILC3 tissue development and/or function.

The Th17 cells in the colon of the D- mice failed to expand and there were significantly fewer Th17 cells in the d10 infected colon compared to D+ mice. 1,25D treatments have been shown to inhibit IL-17 *in vitro* and *in vivo* presumably by direct inhibition of IL-17 transcripts (18, 39, 40). Confirming a direct role of vitamin D on *il-17* mRNA, D- mice over expressed *il17* mRNA in the distal colon following *C. rodentium* infection and the *il17* mRNA was higher in the D- colon than the D+ colon. The higher *Il17* mRNA in the D- colon (Figure 3) could be due to the remaining ILC3 cells in the D- colon producing increased amounts of IL-17 (Supplementary Figure 4A). T cell derived IL-17 was lower in the infected D- colon than the D+ colon. There was no effect of T-VDR KO on host resistance to *C. rodentium*, and therefore it seems unlikely that the failure of Th17 cells to expand following infection is a direct effect of vitamin D on Th17 cells. Instead, the failure of Th17 cells to expand in the D- mice following *C. rodentium* infection could be related to the reduced ILC3 frequencies. ILC3s express MHC class II molecules that present antigens to T cells (41). ILC3 mediated antigen presentation has been shown to induce T cell activation and proliferation of T cells (41). Reduced expansion of Th17 cells following infection of D- mice is likely due to reduced numbers of ILC3 cells important for presenting antigen and inducing proliferation of T cells following activation.

The *Citrobacter*-specific antibody response was lower in D- mice. B cells express the VDR and are vitamin D targets (8). B-VDR KO and VDR KO mice have hyper Ig-E because of the direct role of vitamin D on IL-10 production by B cells (42, 43). Conversely, 1,25D inhibited B cell proliferation, B cell differentiation into plasma cells and IgE production (44). Here B-VDR KO mice had normal *Citrobacter*-specific antibody responses and the B-VDR KO mice cleared the *C. rodentium* infection with the same kinetics as their WT littermates. Based on this data in B-VDR KO mice the effects of vitamin D on antibody responses to *C. rodentium* are likely indirect effects of vitamin D on B cells.

Fewer ILC3, and less IL-22 resulted in the increased susceptibility of D- mice to *C. rodentium* infection. 1,25D treatment of D- mice that commenced at 3wks of age recovered the ILC3 frequencies in the colon. The 1,25D treatments and higher frequency of ILC3 cells improved the resistance of the D- mice to *C. rodentium*. Vitamin D was also needed to expand the infection induced Th17 response. IL-22 treatment of D- mice completely eliminated a need for vitamin D in the protection of D- mice from *C. rodentium* infection. ILC3 cells require vitamin D for either development or expansion and as a source of early IL-22. Vitamin D is a critical regulator of ILC3 cells and as a result D- mice have compromised resistance to *C. rodentium* infection of the gastrointestinal tract.

## Author Contributions

Y-DL, SB, and MC conceptualized and designed the experimental studies. Y-DL, JA, KD, and SB performed the experiments and acquired and analyzed the data. Y-DL drafted the manuscript with the help of MC and SB critically revised the manuscript. All authors approved the publication of the manuscript.

### Conflict of Interest Statement

The authors declare that the research was conducted in the absence of any commercial or financial relationships that could be construed as a potential conflict of interest.
