# Supplementary material for: Vitamin D Is Required for ILC3 Derived IL-22 and Protection From Citrobacter rodentium Infection
Source: Front Immunol. 2019 Jan 22;10:1. doi: 10.3389/fimmu.2019.00001 (PMC6349822; doi:10.3389/fimmu.2019.00001)
Supplement: Supplementary file 1 [file Data_Sheet_1.pdf]

STable 1. Real time PCR primers used

Gene		Primer Sequence
ifn-γ	forward:	TGCATCTTGGCTTTGCAGCTCTTCCTCATGGC
	reverse:	TGGACCTGTGGGTTGTTGACCTCAAACTTGGC
il-17a	forward:	CAGGGAGAGCTTCATCTGTGT
	reverse:	GCTGAGCTTTGAGGGATGAT
il-22	forward:	TGACGACCAGAACATCCAGA
	reverse:	AATCGCCTTGATCTCTCCAC
vdr	forward:	CCCCTTCAATGGAGATTGC
	reverse:	CTGCACCTCCTCATCTGTGA
rorc	forward:	GACAGGGAGCCAAGTTCTCA
	reverse:	CTTGTCCCCACAGATCTTGCA
hprt	forward:	CAGACTGAAGAGCTATTGTAATG
	reverse:	CCAGTGTCAATTATATCTTCCAC
β-actin	forward:	AGAGGGAAATCGTGCGTGAC
	reverse:	CAATAGTGATGACCTGGCCGT

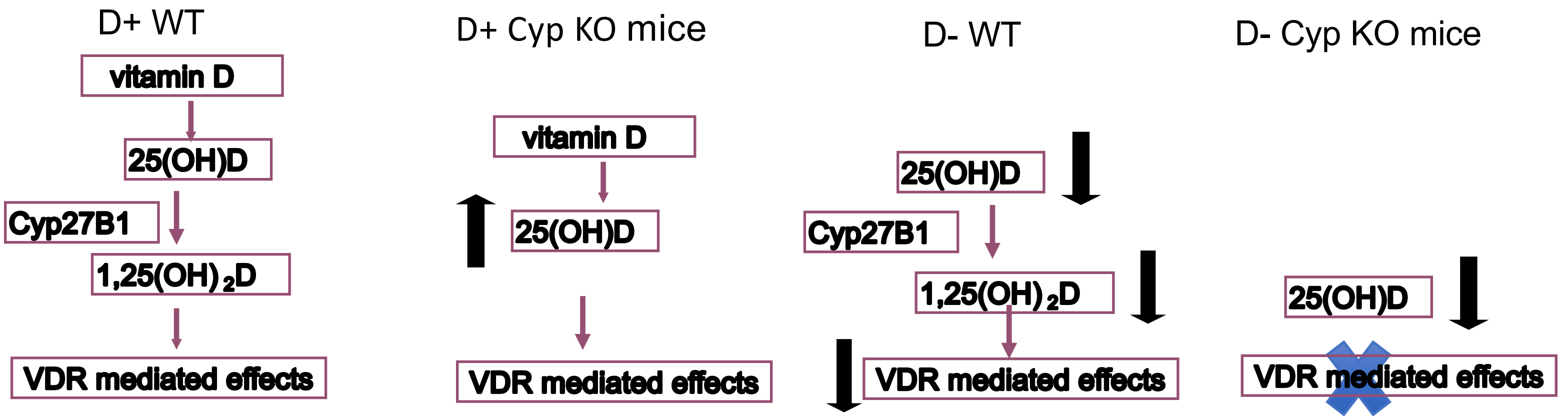

Supplemental figure 1: Cyp27B1 (Cyp) is the enzyme that produces high affinity 1,25(OH)$_2$D from the precursor 25(OH)D. Feeding Cyp knockout (KO) mice diets that contain vitamin D (D+) results in the accumulation of 25(OH)D which is a low affinity ligand for the vitamin D receptor (VDR). Feeding mice diets without vitamin D (D-) greatly reduces but does not eliminate all of the 25(OH)D. D- WT mice produce low levels of 1,25(OH)$_2$D from the residual 25(OH)D. D- Cyp KO mice have low levels of 25(OH)D and no 1,25(OH)$_2$D. D- Cyp KO mice therefore have a complete absence of VDR mediated effects.

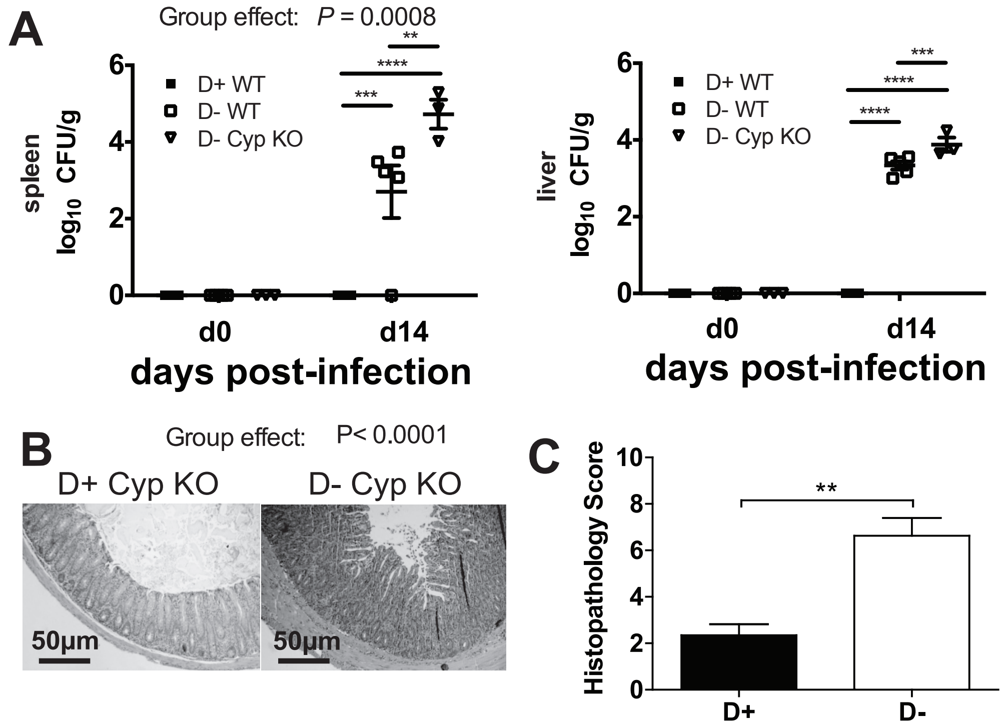

Supplemental figure 2: Systemic *C. rodentium* infection and increased colonic pathology in D- mice.
A) *C. rodentium* CFU/g in the spleen and liver and **B)** colonic histopathology at d14 post-infection. 10x magnification. The D+ Cyp KO section histology score = 2 and the D- Cyp KO histology score shown = 6.
C) Combined histological scores of the distal colon at d14 post-infection, n=4 mice/group. Significance was determined using two-way ANOVA with Bonferroni post-hoc tests (A) and two-tailed Student's t test (C) ** P<0.01, ***P<0.001.

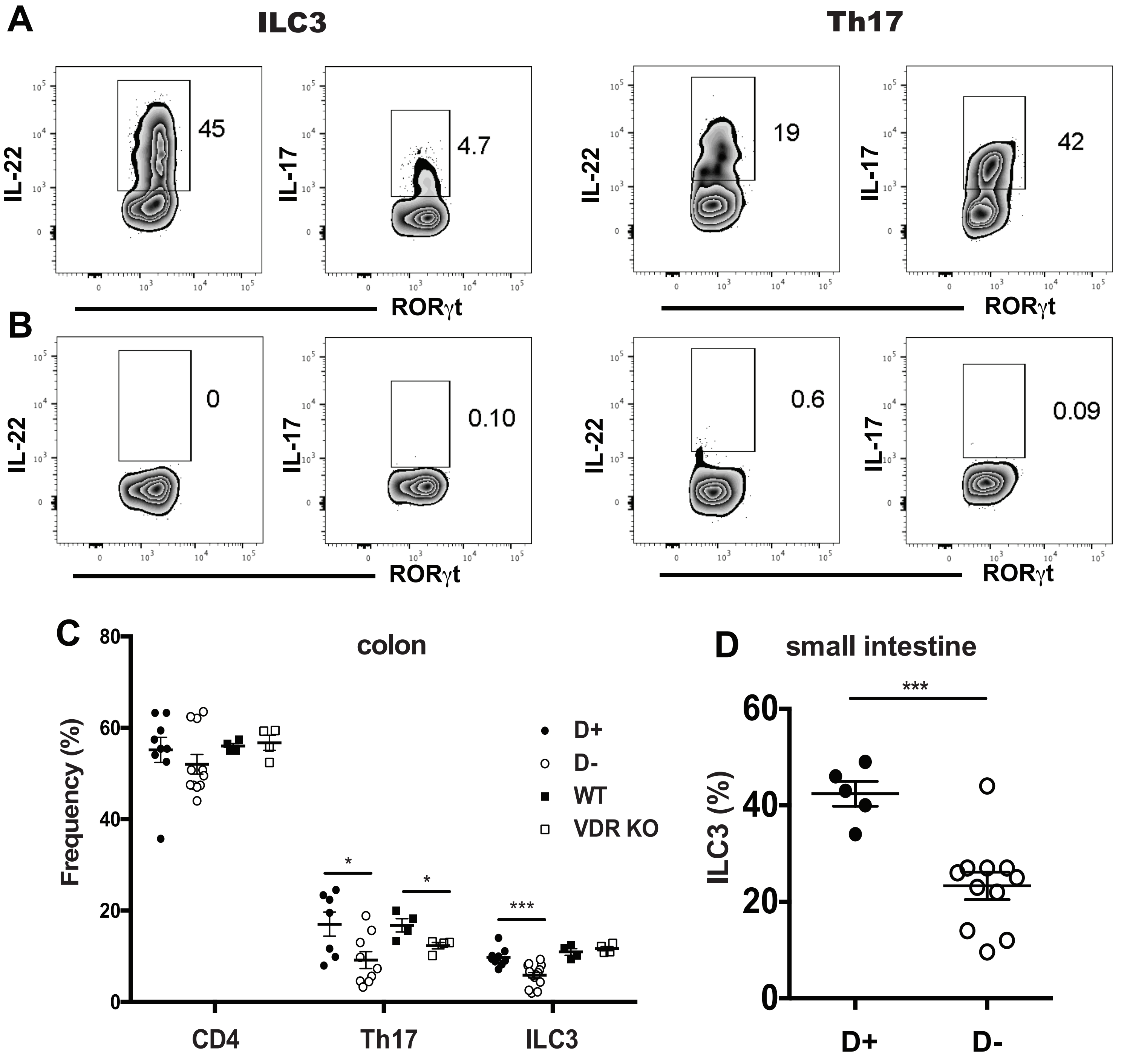

Supplementary figure 3. Frequecies of Th17 and ILC3 in the colon and small intestine of D- and VDR KO mice. A) and B) Reprentative Intracellular cytokine staining plots of IL-22 and IL-17 in colonic ILC3s and Th17 cells. **C)** Frequencies of Th17, and ILC3 in the colon of D+, D-, WT and VDR KO mice. Data shown are representative or combined data of two independent experiments with n=4-9/group. **D)** Frequencies of ILC3 cells in small intestinal LPL of D+ and D- mice. Data shown are combined data of two independent experiments with n=5-11/group. Significance was determined using two-tailed Student's t test * $P<0.05$,

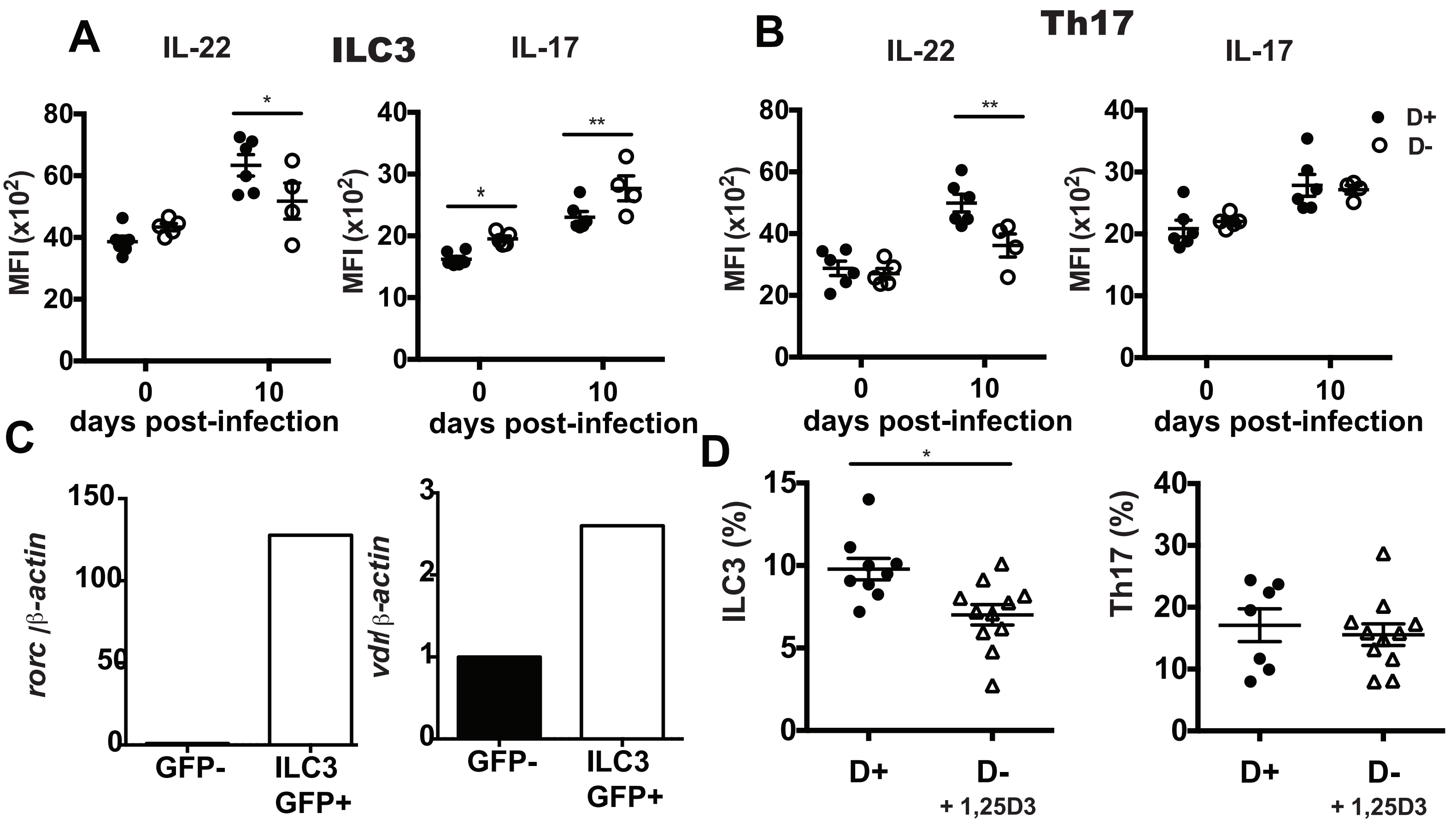

Supplementary figure 4. MFI and effect of 1,25D on immune cells in the colon. Mean fluorescent intensity (MFI) of IL-22 and IL-17 staining in **A)** ILC3 or **B)** Th17 cells in the colon of D+ and D- mice. Values are mean ± SEM of two combined experiments and n= 4-6 mice/group. Significance was determined using two-way ANOVA with Bonferroni post-hoc tests. D- mice were treated with 1,25D beggining at 6 wks of age. Frequencies of **C)** RORγt+ ILC3 cells express higher *vdr* mRNA in the colon than other ILCs. Relative mRNA expression for *rorc* and *vdr* in FACS-purified RORγt+(GFP+) and GFP- ILCs isolated in the intestine of Rag1 KO mice. **D)** ILC3 and Th17 in the colon. Values are mean ± SEM from 2 independent experiments and n= 8-11 mice/group. Two-tailed Student's t,* $P<0.05$.
